# Transcultural Psychiatry: Cultural Difference, Universalism and Social Psychiatry in the Age of Decolonisation

**DOI:** 10.1007/s11013-021-09719-4

**Published:** 2021-04-27

**Authors:** Ana Antić

**Affiliations:** Department of English, Germanic and Romance Studies, University of Copenhagen, Copenhagen, Denmark

**Keywords:** Transcultural psychiatry, Cultural difference, Social psychiatry, Decolonization

## Abstract

In the mid-twentieth century, in the aftermath of WWII and the Nazi atrocities and in the midst of decolonisation, a new discipline of transcultural psychiatry was being established and institutionalised. This was part and parcel of a global political project in the course of which Western psychiatry attempted to leave behind its colonial legacies and entanglements, and lay the foundation for a more inclusive, egalitarian communication between Western and non-Western concepts of mental illness and healing. In this period, the infrastructure of post-colonial global and transcultural psychiatry was set up, and leading psychiatric figures across the world embarked on identifying, debating and sometimes critiquing the universal psychological characteristics and psychopathological mechanisms supposedly shared among all cultures and civilisations. The article will explore how this psychiatric, social and cultural search for a new definition of ‘common humanity’ was influenced and shaped by the concurrent global rise of social psychiatry. In the early phases of transcultural psychiatry, a large number of psychiatrists were very keen to determine how cultural and social environments shaped the basic traits of human psychology, and ‘psy’ practitioners and anthropologist from all over the world sought to re-define the relationship between culture, race and individual psyche. Most of them worked within the universalist framework, which posited that cultural differences merely formed a veneer of symptoms and expressions while the universal core of mental illness remained the same across all cultures. The article will argue that, even in this context, which explicitly challenged the hierarchical and racist paradigms of colonial psychiatry, the founding generations of transcultural psychiatrists from Western Europe and North America tended to conceive of broader environmental determinants of mental health and pathology in the decolonising world in fairly reductionist terms—focusing almost exclusively on ‘cultural difference’ and cultural, racial and ethnic ‘traditions’, essentialising and reifying them in the process, and failing to establish some common sociological or economic categories of analysis of Western and non-Western ‘mentalities’. On the other hand, it was African and Asian psychiatrists as well as Marxist psychiatrists from Eastern Europe who insisted on applying those broader social psychiatry concepts—such as social class, occupation, socio-economic change, political and group pressures and relations etc.—which were quickly becoming central to mental health research in the West but were largely missing from Western psychiatrists’ engagement with the decolonising world. In this way, some of the leading non-Western psychiatrists relied on social psychiatry to establish the limits of psychiatric universalism, and challenge some of its Eurocentric and essentialising tendencies. Even though they still subscribed to the predominant universalist framework, these practitioners invoked social psychiatry to draw attention to universalism’s internal incoherence, and sought to revise the lingering evolutionary thinking in transcultural psychiatry. They also contributed to re-imagining cross-cultural encounters and exchanges as potentially creative and progressive (whereas early Western transcultural psychiatry primarily viewed the cross-cultural through the prism of pathogenic and traumatic ‘cultural clash’). Therefore, the article will explore the complex politics of the shifting and overlapping definitions of ‘social’ and ‘cultural’ factors in mid-twentieth century transcultural psychiatry, and aims to recover the revolutionary voices of non-Western psychiatrists and their contributions to the global re-drawing of the boundaries of humanity in the second half of the twentieth century.

## Introduction

In the mid-twentieth century, in the aftermath of the WWII atrocities and in the midst of decolonisation, a new discipline of transcultural psychiatry was being established and institutionalised. This was part and parcel of a global political project in the course of which Western psychiatry attempted to leave behind its colonial legacies and entanglements, and lay the foundation for a more inclusive, egalitarian communication between Western and non-Western concepts of mental illness and healing (Bullard 2007). In this period, the infrastructure of post-colonial global and transcultural psychiatry was set up, and leading psychiatric figures across the world embarked on identifying, debating and sometimes critiquing the universal psychological characteristics and psychopathological mechanisms supposedly shared among all cultures and civilisations.

The article will explore this psychiatric, social and cultural search for a new definition of ‘common humanity’. In the early phases of transcultural psychiatry, a large number of psychiatrists were very keen to determine how cultural and social environments shaped the basic traits of human psychology, and ‘psy’ practitioners and anthropologist from all over the world sought to re-define the relationship between culture, race and individual psyche. Most of them worked within the universalist framework, which posited that cultural differences merely formed a veneer of symptoms and expressions while the universal core of mental illness remained the same across all cultures. Even in this context, which explicitly challenged the hierarchical and racist paradigms of colonial psychiatry, the founding generations of transcultural psychiatrists from Western Europe and North America tended to conceive of broader environmental determinants of mental health and pathology in the decolonising world in fairly reductionist terms—focusing almost exclusively on ‘cultural difference’ and cultural, racial and ethnic ‘traditions’, essentialising and reifying them in the process, and failing to establish some common sociological or economic categories of analysis of Western and non-Western ‘mentalities’. Importantly, the emergence of transcultural psychiatry corresponded chronologically with the rise to prominence of social psychiatry in the Western world, but the two disciplines tended to remain separate throughout the second half of the twentieth century: even though they were deeply interconnected, they were marked by significant differences in their methodologies, core preoccupations and conceptual frameworks.

For both disciplines, the experiences of the Second World War was crucial and formative, and, while both had roots in the pre-war period, they experienced unprecedented growth and rose to prominence within the broader profession of psychiatry in the aftermath of 1945 (Wu 2021). For the development of the movement of social psychiatry, the experience of wartime psychopathologies and ‘war neuroses’ played an enormous role, as a number of practitioners moved beyond organicist psychiatric paradigms and began to explore alternative aetiologies, focusing in particular on environmental causes of mental illness. Social psychiatry thus increasingly emphasised ‘the interactions between the individual and social genesis of [mental] disease’ (Haack and Kumbier 2012). In the words of one of the fathers of British social psychiatry, Aubrey Lewis, this novel subdiscipline explored the aetiological role of ‘culture’ conceived in its broadest sense as ‘social heritage.’ Such psychiatric research included investigations into the widest range of sociological (‘economic, ethnic and cultural’) categories which defined the ‘social context of illness’.^1^ However, social psychiatry tended to develop primarily in the context of a shared culture between therapists and patients (Neve 2004). When these concerns with environmental factors and socio-cultural contexts of mental illness were transferred to transcultural settings and applied to psychiatric encounters outside the Western world, the notions of ‘culture’ and ‘social heritage’ tended to be defined much more narrowly, and the array of social scientific categories which informed psychiatric work was generally significantly decreased.

In many ways, transcultural psychiatry should have functioned as a sub-field of the social psychiatry movement, especially given the transdisciplinary focus of cross-cultural ‘psy’ practitioners on exploring cultural differences as determinants of mental illness.^2^ In fact, in the early post-war planning for global mental health projects at WHO, social and cross-cultural psychiatric research were deeply interrelated: the new discipline of international psychiatry saw comparative cross-cultural investigations as an organic element of its search for environmental stressors and causes of mental illness (Wu 2015). Transcultural psychiatry was also fundamentally shaped by the extreme violence of the Second World War, and became an essential part of the postwar social and psychological reconstruction and search for peace as a discipline which promised to better understand and facilitate transcultural communication and collaboration (Chisholm 1946; Bains 2005). In the era of decolonisation, moreover, transcultural psychiatry sought to define a set of universal psychological characteristics which would make such cross-cultural understanding and harmony possible (Heaton 2013). However, in the Western world, the long shadow of colonial psychiatry partly shaped the new post-colonial profession’s prioritisation and essentialisation of ‘cultural difference’, and this often worked to set it apart from mainstream social psychiatry developments.^3^

In fact, it was mainly African and Asian psychiatrists as well as Marxist psychiatrists from Eastern Europe who insisted on applying those broader concepts to describe their patients’ rich social worlds. In this way, some of the leading non-Western psychiatrists sought to establish the limits of psychiatric universalism, and challenge some of its Eurocentric and essentialising tendencies. Even though they still subscribed to the predominant universalist framework, these practitioners invoked social psychiatry to draw attention to universalism’s internal incoherence, and sought to revise the lingering evolutionary thinking in transcultural psychiatry. They also contributed to re-imagining cross-cultural encounters and exchanges as potentially creative and progressive rather than pathogenic (Ayonrinde and Pringle 2020; Antic 2019).

Therefore, the second half of the twentieth century saw a global re-drawing of the boundaries of humanity through complex and involved discussions in the discipline of transcultural psychiatry. In the course of this process, the definitions of ‘social’ and ‘cultural’ factors shifted and overlapped in multiple ways, and the article explores the complex politics of these changing definitions.

The article is structured around thematic clusters rather than chronologically, and it focuses on the most important problems in the history of transcultural psychiatry. It begins by exploring the dominance of narrowly defined ideas of cultural difference within the universalist framework of Western global and transcultural psychiatry in the aftermath of the Second World War. It then proceeds to examine how this paradigm was challenged from different corners. Firstly, psychiatrists from the decolonising world and socialist Eastern Europe reconsidered the complex interaction between social and cultural factors, offering a nuanced and innovative critique. Secondly, an alternative, postcolonial cultural relativist approach, exemplified by the Fann hospital experiment, sought to downplay the importance of universal and cross-cultural models of human psychology. The final section demonstrates that, because of its unresolved internal incoherence and tendency to essentialise cultural traits, transcultural psychiatry could not properly grapple with its main concern—the issue of cross-cultural encounters, exchange and understanding—and struggled to move beyond the colonial interpretations of cross-cultural contact as a major source of mental pathology.

The article employs the concepts of ‘Western’ and ‘non-Western’ to differentiate between psychiatrists and anthropologists from different parts of the world (in particular, ‘non-Western’ is used to describe those practitioners who came from outside the West European and North American centres of medical knowledge production, while ‘Western’ protagonists are those originating primarily from the former colonial metropoles, and the USA and Canada). The binary, of course, is problematic and this article by no means argues that it should be understood as absolute—not least because it homogenises the ‘non-Western other’ and defines it negatively in relation to the ‘West’ (Hutchings 2011; Hall 2002). Moreover, it tends to essentialise the link between knowledge and geographical or cultural origins, and cannot accommodate the complexity of networks and connections which developed across the imagined boundary. I primarily use these appellations as a shorthand device to emphasise that some professionals and practitioners functioned outside the dominant centres of hegemonic power (which very much remained so both before and after the decolonisation processes), while others had relatively privileged access to a variety of educational, research and managerial opportunities available in the global North. This fundamental difference shaped their experiences and broader professional roles and outlook. Of course, as we will see below, none of the ‘non-Western’ protagonists of this story was so in any absolute way: most professionals from Eastern Europe or the decolonising world either received part of their education in Western Europe, or became permanently involved in ‘Western’ organisations and structures of power (Pringle 2019). The very instability of their position and cultural and professional belonging—their capacity for complex crossovers—fundamentally contributed to the innovative and critical nature of their interventions, as will become clear in the rest of the article.

### Supremacy of the Cultural

‘May I say here that I have visited many countries in the world and I don’t believe there are fundamental differences or basic differences between anything that I heard in Africa and the things you find in other countries, except naturally in the cultural determinants of some symptoms of illness,’ pronounced John Rees, head of the WFMH, at the organisation’s conference in 1959 (WFMH 1959: 29–30). Throughout the late 1950s and 1960s, some of the leading global psychiatrists insisted on downplaying the importance of any cultural differences for psychiatric practice and nosologies. US transcultural psychiatrist Ari Kiev, who authored a seminal volume on ‘primitive psychiatry’, firmly established a core similarity between Western and non-Western therapeutic practices in terms of their core concepts, the emotional involvement of both recipients and therapists, and the responses they aim to elicit or deliver. Kiev concluded that ‘It seems not unlikely then that mental illness is manifested in certain basic structural mechanisms and processes that recur together with certain regularity in the different clinical syndromes, providing a substratum on top of which the different cultures impose differences in content. There is little evidence to suggest that mental illnesses differ from culture to culture in any different way’ (Kiev 1964: 19). Edward Margetts, Canadian psychiatrist heading the Mathari hospital in Kenya, who appeared to believe that the ‘primitive’ peoples of Eastern Africa possessed bizarre cultures and mentalities, fundamentally different from those of the ‘civilised’ world, still confessed that ‘in my studies of African psychiatry, I have accepted diagnoses and treatments more or less as practiced in the civilized areas… The results in general are quite comparable to those one would expect in a civilised population’ (Margetts 1958b). At this time, even John Colin Carothers, Margetts’ infamous Kenya-based colleague, whom Eric Linstrum has referred to as a ‘champion of settler views on racial inferiority’, sought to explain his idea of the Africans’ supposed inability for rational thinking and ‘frontal lobe laziness’ not in terms of heredity and innate deficiencies but as caused by environmental—and thus changeable—factors (Linstrum 2016). Carothers was controversially promoted to a WHO consultancy in 1952, and his move away from the notion of hereditary racial difference was partly in line with the universalist sentiments of his new host organisation, even though his unfounded and simplistic interpretations were widely challenged by post-war psychiatrists and anthropologists.^4^ For early transcultural psychiatrists, therefore, the universalist framework meant that the core of mental illnesses remained the same across different cultures and societies, while cultural differences simply constituted a ‘veneer’ of symptoms and expressions.(Margetts 1958b: 581). However, despite this apparent dismissal of the fundamental significance of ‘culture’, many transcultural psychiatrists in the second half of the twentieth century tended to formulate overly culturalist explanations of mental disorders in the decolonising world, disregarding a variety of social, economic and political factors, and exoticising and reifying the very idea of cultural difference in the process.

Margetts concluded that any differences in expressions of mental illness in East African societies were mainly due to rather narrowly defined cultural phenomena: ‘firstly tribal custom and law, secondly detribalisation, mixing of races and displacement, and thirdly to attitudes taken up by simple uneducated Africans, as a result of the so-called “nationalism” fostered by politicians and organizers’ (Margetts 1958a: 1). In other words, for Margetts—a sceptic when it came to the local population’s political ability and capacity for self-governance—the only psychopathological factor related to the Kenyans’ broader socio-political environment and experiences was anti-colonial and nationalist political activism, which he claimed fostered highly deleterious negative emotions such as ‘distrust and suspicion.’ Other than that, his analysis zoomed in on exclusively cultural concepts—customs and ‘tribal’ regulations, as well as the supposedly psychopathological effects of cultural contacts, exchange and mixing, and of cultural change (‘detribalisation’, or ‘displacement’) in African societies (Margetts 1958a).

Even when his publications were entirely pragmatically oriented, they betrayed his belief in the absolute theoretical and clinical primacy of narrowly defined cultural influences. For instance, Margetts’ comprehensive guidelines for anthropological-psychiatric research in East Africa included a variety of cultural factors and phenomena under the heading ‘Psychopathology and causation’—‘magical numbers’, ‘religion’, ‘amulets’, ‘cannibalism’, ‘sacrifices’, ‘possession’, and ‘witchcraft,’ Some of his other headings referred to ‘curses’, ‘cults’, ‘magical practitioners’, and ‘charms, amulets and fetishes, but none of these concepts encouraged or enabled practitioners to take clinical account of broader socio-economic circumstances, sociological categories or political change.’ (Margetts, 1958a: 1–12). His analysis reduced the complex psychological effects of the massive social and economic dislocations in post-independence East Africa to ‘acculturation’ ,^5^ a late colonial concept almost entirely focused on ‘cultural incommensurability’ between African and European populations (Margetts 1960). It is striking that such an extraordinarily reductive conceptualisation of ‘African environments’ could co-exist in Margetts’ worldview (and in early transcultural psychiatry as a whole) with rather strong professions of universalism and universal applicability of Western categories. It is difficult to see how West European and Kenyan forms of ‘madness’ could be compared given that the categories used to analyse mental illness in these two parts of the world never overlapped in any way.

This trend continued well after Margetts’ explorations. In the first postwar decades, some of the most prominent Western transcultural psychiatrists often insisted on cultural and ethnic specificities at the expense of other social factors—such as class or poverty—which played an important role in shaping both Western and non-Western conceptualisations of mental health and illness. For instance, Eric Wittkower, another founding figure of the new discipline of transcultural psychiatry and the first editor its core journal *Transcultural Psychiatric Research Review*, offered a conference presentation about ‘recent developments in transcultural psychiatry’ at the 1965 Ciba Congress of transcultural psychiatry, which gathered almost all the most important figures in the field (Bains 2005).^6^ A few audience members questioned his omission of any substantial discussion of the importance of sociological categories—class, education, age, gender, occupation—in cross-cultural psychiatric and anthropological research. As Alexander Leighton pointed out in his comment, in Western multicultural societies such as Canada or the US, social class and other social categories played a very important role in mental health research. However, as Wittkower’s presentation and curt responses illustrated, when it came to non-Western societies, transcultural psychiatry often tended to focus on cultural, racial and ethnic traditions, failing to establish some common sociological or economic categories of analysis of Western and non-Western ‘mentalities’ (Wittkower 1965).

Outside the European psychiatric profession, local psychiatrists from the former colonies often subscribed to this unwritten theory about the unchallenged supremacy of the cultural. For instance, British-educated Gambian psychiatrist Emmanuel Forster wrote a long and informative treatise on mental health practices and values in Ghana, the country in which he practiced and served as the first Chair of Psychiatry, and focused on the complex psychological profiles of ‘Africans in transition’—those Ghanaian patients whose everyday lives and value systems were disrupted by the massive social, economic and political changes tied up with the onset of independence, modernisation and industrialisation in West Africa (Asare 2012). Forster’s account was multifaceted and nuanced in parts, and touched on a variety of contextual factors, such as the sociological pressures of labour migration, anxieties linked to the turbulent politics of self-determination and nation-building, and disintegration of social units. However, his conclusion effectively marginalised the etiological or explanatory significance of these factors, and Forster ended by claiming that ‘as a broad base line, the African’s psychological illness is [primarily] linked to his peculiar beliefs in witchcraft, juju, superstitions, and the all-powerfulness of the supernatural’ (Forster 1962: 44). It naturally followed from this, moreover, that any psychiatrist or psychotherapist who had ‘some understanding of the cultural background of the patients,… their superstitions, beliefs and fears’ should be able to practice with effectiveness and success in African mental health settings. It was this ‘traditional’ cultural knowledge that was deemed essential here, rather than any deeper familiarity with the rapidly changing social and political conditions in Ghana (Forster 1962: 44).

This trend continued well after the 1960s, and marked the history of the discipline throughout the second half of the twentieth century, perpetuating a fundamental contradiction at its intellectual heart and preventing a genuine comparison between Western and non-Western experiences of mental illness. The late colonial evolutionary theory persisted in transcultural psychiatry in different guises.^7^ One of its more imaginative reformulations came from the writings of Britain’s foremost cross-cultural psychiatrist and one of the leaders of the UK section of the International Pilot Study of Schizophrenia (IPSS) Julian Leff. The IPSS was a WHO-funded project, whose scope and ambition were unprecedented in global and transcultural psychiatry thus far: starting in 1966, it involved the establishment of nine field research centres around the globe, and engaged in clinical research with patients diagnosed with schizophrenia.^8^ This massive operation aimed to explore whether schizophrenia might be a universal psychiatric category and how cultural differences affected the symptoms and experience of the disease, as well as to standardise diagnostic criteria, classifications and therapies (Sartorius 1972: 422–425). The IPSS was a milestone in the post-war search for universal dimensions of human psychology, and it established transcultural psychiatry as a global research and clinical field.

Leff was one of the most prolific IPSS participants, continuing to publish important research about its long-term legacies. One of Leff’s most prominent contributions is his theory, based in large part on IPSS materials and experiences, of the development and differentiation of ‘languages of emotion.’ In it, Leff argued that an extensive and differentiated vocabulary concerning emotional states is only characteristic for developed countries of the Western world. Leff indicated a ‘scheme’ of an ‘evolutionary process in the dawning awareness of the psychological experiences of unpleasant emotion’, according to which he classed most non-Indo-European languages as ‘living fossils’, left at an earlier stage of social and psychological development. Speakers of these languages, according to Leff, normally ‘lacked words for depression and anxiety and instead [possessed] words for the bodily experiences of emotion which are relatively undifferentiated’ (Leff 1981: 45–46). Leff’s scheme was based on the idea of a linear historical process in the course of which people’s consciousness of their own psychological experiences developed and grew ever more sophisticated.

Leff’s linear interpretation explicitly placed the non-Western world in an altogether separate historical stage of linguistic and psychological development, and further argued that in traditional societies, where the ‘cultural focus’ was on the group rather than individual, ‘there is little opportunity to explore the emotional aspects of relationships…. In traditional societies, where relationships are more or less stereotyped, emotions remain unexplored and undifferentiated’ (Leff 1981: 72). The move towards the individual in the West, on the other hand, was the main ‘motive force behind the increasing differentiation of emotions and the expansion of the lexicon of emotions to allow the new refinements of experience to be communicated.’ In other words, Leff’s reading of IPSS materials portrayed ‘Western’ minds as more complex and sophisticated, as a result of long-term social and cultural historical processes, which possessed an ‘incalculable momentum’ compared to the developing societies. Colonial psychiatrists and psychoanalysts often ascribed extreme psychological simplicity and lack of differentiated personalities to Asians and Africans. Leff appeared to argue that the IPSS findings in the field research centres outside the Western world (such as in Colombia, Nigeria, India etc.) confirmed those earlier theories (Leff 1973).

Leff’s theory was not a far cry from psychiatrists Benedict and Jacks’ 1954 conclusion that the clinical picture of ‘primitive’ psychosis was ‘a poor imitation of European forms’ due to its lack of richness and complexity (Benedict and Jacks 1954: 377). In late colonial and early transcultural psychiatric discussions, this reported poverty of language signified a more profound mental limitation: Margetts, opined that ‘because of limitations of expressiveness of the languages concerned and of their lack of “education” and understanding, most natives find it difficult or impossible to describe themselves accurately even in their own language, let alone a secondary one.’ (Margetts 1958b: 679–683).

But it is important to note that such reductive culturalist explanations were not characteristic solely for Western psychiatrists: Forster tackled the issue of linguistic limitations in psychiatric encounters in West Africa, emphasising first that language problems mainly emerged because patients spoke a variety of different idioms and not all were conversant in English. But he quickly abandoned this explanation which hinged on the linguistic mismatch between patients and practitioners and developed his argument in a value-laden direction, adding the ‘primitive’ simplicity of West African languages as another issue: ‘because of the limitations of expressiveness in the language concerned, and the lack of education and understanding, most patients find it difficult or impossible to describe their symptoms accurately even in their own language’—an observation perhaps difficult to prove given that Forster himself had admitted to not understanding many of these languages in the first place. In particular, and in agreement with Leff, Forster emphasised his patients’ difficulty with ‘[verbalizing] certain subjective emotional reactions’ (Forster 1962).

In the 1980s, Leff’s contention came under criticism from the anthropological corner, as an excellent example of what Arthur Kleinman later referred to as a ‘category fallacy’—an assumption that mainstream psychiatric categories were not highly culturally specific products of Western epistemologies and worldviews but expressed something of the universal core of mental illnesses, and that those same categories and diagnoses could be recognised in other parts of the world, hidden under layers of cultural modifications and specificities (Kleinman 1977).^9^ Indeed, Leff took a Western list of emotional concepts to non-Western communities, and concluded, unsurprisingly, that they had a less differentiated set of terms for those concepts than the English language. The fallacy was, however, in the next step in Leff’s argumentation: this did not necessarily mean that individuals in those communities were themselves less psychologically differentiated or emotionally sophisticated, but that their definitions and experiences of selfhood may have differed significantly from the Western model, and that their ‘languages of emotions’ likely focused on a different range of internal mental states (Myers 1979).

Importantly, even though the ‘somatization’ hypothesis was often discussed and tested by a variety of psychiatric and anthropological experts, it was striking that Leff chose to interpret it almost exclusively a result of cultural differences and specificities. As Roland Littlewood has subsequently argued, such reductive cultural explanations ‘[oversimplified] the multifactorial aetiology of the diseases studied’ (Littlewood and Lipsedge 1999: 269). Discussing the same issue, for example, psychologist Bal emphasised the sociological background of patients and the social context of psychiatric consultation, arguing that ‘working-class Asian patients are likely to use a mode of communication [physical complaints] which they believe will be acceptable to the doctor, and one which does not involve blaming family members for their distress’ (Bal 1987). Following his ethnological and clinical research in Uganda, anthropologist and psychiatrist John Orley has argued similarly that a tendency to frame distress in the ‘language of body illness’ often stems from the patient’s ‘desire to express his illness in what he thinks are terms acceptable to western medicine’ (Orley 1970: 50). Moreover, in her examinations of Punjabi patients in Bedford, Krause also noted the predominance of physical complaints but emphasised their multiple meanings and implications. Rather than viewing such bodily complains as symptoms of patients’ underdeveloped idioms of psychological suffering, she concluded that they ‘[embodied] complex relations between external events, subjective experiences and personal selfhood, autonomy and control’ (Krause 1989: 563–565). In other words, they were no less sophisticated than Western expressions of emotional distress.

Despite the proclaimed psychiatric universalism and its tendency to disregard the importance of cultural differences as merely occluding the universal core of illnesses, leading figures such as Leff paradoxically focused on perceived cultural and civilizational distinctions as essential determinants of mental health experiences, often missing an opportunity to explore broader sociological and economic circumstances which shaped the lives of both Western and non-Western patients.

### Social Psychiatry Outside the West

As post-war transcultural psychiatry engaged with the project of reconstructing the relationships between individual, culture, race and species in the midst of major global political and social transformations, universalism proved to be a complex and internally incoherent intellectual framework, used to signal liberal and anti-colonial sentiments, but also, as we saw, combined colonial tropes with a reification of cultural difference. It was researchers and psychiatrists from outside the Western centres of psychiatric knowledge production who struggled with the universalist paradigm the most, and strove to emphasise its value in countering colonial hierarchies while grappling with its Western ethnocentrist tendencies.

In order to counter racist colonial psychiatry’s notions of hierarchical fundamental difference between Europeans and the ‘African mind’, Nigeria’s most important psychiatrist Thomas A. Lambo worked to reformulate the dominant concepts of boundaries of cultural units and of relationships between them, and aimed to ‘produce research arguing for the basic universal similarity of human psychology, irrespective of race, religion, ethnicity or geography’ (Heaton 2013: 52).

Starting with his dissertation research at the University of Birmingham, Lambo identified Carothers’ arguments about the inferiority of the ‘African mind’ as ‘dangerous to scientific thinking.’ Lambo endeavoured to prove unfounded Carothers’ claims that ‘African backwardness and the occurrence of “primitive psychosis” can well be linked to frontal idleness’, and used his schizophrenia research on the Yoruba people in Nigeria to demonstrate that ‘the nature of men is the same, what divides them is their custom.’ While Lambo was fully aware of the myriad cultural nuances and factors relevant to psychiatric research, his argument in favour of universality hinged on the idea that mental distress in Africa and in the Western world shared fundamental mechanisms which could be explained ‘in terms of common psychodynamic formulations’ (Lambo 1955). The political implications of Lambo’s work underscored the liberal and progressive origins of such psychiatric universalism in the de-colonizing territories. At the same time, however, later on in his career Lambo urged the psychiatrists from the Western world to ‘decontaminate themselves intellectually from Freudian and neo-Freudian theories’, which represented ‘at their best a spectrum of possible ideas emanating from Hellenic and Judaeo-Christian culture and tradition’, and were not particularly helpful when dealing with mental patients from different cultural traditions, or when describing their psyches and psychological processes (Heaton 2013: 73).

Psychiatric practitioners from the decolonising territories were in a particularly complex position, threading a thin and sensitive line between humanistic universalism, which could easily veer into a reinforcement of Eurocentric models of the mind, and insistence on the importance of culturally specific influences, a viewpoint that at times seemed dangerously close to colonial ethnopsychiatry. Tigani El-Mahi, the legendary Sudanese psychiatrist, faced a similar problem as Lambo, although el Mahi appeared to insist more forcefully on the significance of culturally specific value systems and traditions. In his talk at a WFMH meeting, he qualified the value of psychiatric universalism and used his discussion of the process of diagnosing and treating patients in different cultures to reaffirm the importance of nuanced cultural understanding and affinity. If a therapist was to be successful, he or she needed to be in agreement with patients on the causes and treatments of mental distress. For a psychotherapy to be effective in any culture or society, it needed to provide an aetiological theory which could be in line with the socio-cultural context/assumptions, and accepted by the society at large. It was only in such a culturally sensitive and nuanced context that positive psychological responses could be expected from (psychiatric) patients (El-Mahi 1960). A mental health worker … ‘must support the values of his community and of his day…you must love that community and you must love its culture. Out of our love for our culture, we have been able to understand that many of the demoniacal states, the possessional states, have extremely valuable therapeutics for the patient himself… a really therapeutic basis’ (WFMH 1959: 20).Moreover, he warned Western practitioners that ‘psychiatry is inseparable from the community and must follow it as a shadow.’ Pushing against both colonial hierarchical thinking and post-colonial patronising references to backwardness and primitivism, El-Mahi urged his colleagues to see Africans as ‘adult humanity conscious of its own wisdom, penetrated by its own universal philosophy’ (El-Mahi 1960).

Non-Western transcultural psychiatrists also insisted on the importance of sociological and socio-economic analysis instead of focusing on exclusively cultural interpretations of mental distress. In a 1975 interview with Philip Singer, Lambo argued passionately in favour of working with traditional healers, depicting them as promoters of social psychiatry in the African world: ‘And this, in fact, is one of the tremendous human qualities of the traditional healers. That they can listen, they really have tremendous interest, emotional empathy, and relationship’ (Singer 1977). In fact, the rise of social psychiatry in the Western world proved to be very important for the development of cross-cultural global psychiatry. Some of the leading non-Western transcultural psychiatrists relied on the core arguments of the social psychiatry movement in order to explain the importance of cross-cultural mental health research as well as to test the limits of psychiatric universalism. For instance, the University of Ibadan’s Department of Psychiatry submitted a funding application for a local project focused on schizophrenia research, as the most common mental health affliction among Africans, and only proposed to explore the ‘high rate of population growth, increased mobility of the population, rapid socio-economic change [and] the changing age structure’ as the core factors contributing to an growing incidence of schizophrenia in the developing world.^10^ In his writings and research, Lambo emphasised the importance of conceiving of African patients as ‘social beings’ (rather than representatives of alien and illegible cultures). Lambo viewed ‘the new medical problems of Africa’ as a purview of social medicine and social psychiatry primarily, calling for the ‘establishment of a complete social morphology and a more refined analysis of the complex life of African social institutions,’ instead of simply accepting that psychological problems of Africans stemmed from a clash of traditional societies/individuals with some incompatible European values (Lambo 1960).

In an excellent—and typical—example of this move from reductivist cultural to more broadly comparable sociological explanations, a group of Indian psychiatrists based at the University of Lucknow in Uttar Pradesh, explored the role of social and cultural factors for the Indian population’s the perception and experiences of mental illness. Brij Sethi, Swadesh Sachdev and Devika Nag first focused on some ‘deeply entrenched traditions’ of the Indian society, i.e. the widespread belief in reincarnation and in the force of ‘karma’, which reportedly produced feelings of helplessness among Indians who faced mental suffering as well as their need to rationalise illness rather than seeking psychiatric help. The three psychiatrists’ focus on cultural differences and deeply ingrained non-Western beliefs, however, did not last long: while offered as one possible explanation, it did not form the core of the article’s interpretive frameworks. Instead, Sethi and his colleagues quickly moved on to explore cases of individual patients, none of which involved any mentions of reincarnation or karma; on the contrary, they were all explained with reference to significant psychological effects of social changes in family structure and family relationships (linked to India’s modernisation and industrialisation), evolving models for gender and marital roles, and shifting relationships between younger and older generations and their expectations (Sethi 1965). All these themes and categories could easily be explored in a variety of Western and non-Western societies, and could thereby link Indian psychology (or ‘psyche’) to broader global trends rather than setting it apart in its cultural difference. Moreover, this approach depicted India as a dynamic and constantly evolving society—‘India is in the midst of industrial revolution, … and greater opportunities in industry and defense have provided people with broader horizons than ever before’—a far cry indeed from images of archaism and pathological resistance to change so common in the Western transcultural psychiatry of the time (Sethi 1965: 449).

But there was another important yet systematically overlooked site of knowledge production in the field of global mental health. In socialist Eastern Europe, Marxist transcultural psychiatry engaged in original research projects, developed an alternative theory of non-Western universalism and moved away from reductive and narrow culturalist explanations. Scholarship on global transcultural psychiatry has so far completely ignored socialist and East European perspectives and contributions, even though they offered an original approach to bridging differences between the European and decolonising worlds. In the context of the Cold War and in the midst of decolonisation, socialist psychiatrists and anthropologists from Eastern Europe became increasingly involved in professional networks of socialist globalisation, and took part in technical aid and exchanges missions in the global South. In the course of these exchanges, socialist psychiatry contributed to global discussions about cross-cultural definitions of mental health and disease. For one of the leading East European and Yugoslav transcultural psychiatrists, Vladimir Jakovljevic, for instance, comparisons between Africa and Eastern Europe came naturally and effortlessly, as he discussed—both obliquely and directly—similarities between sub-Saharan and Balkan ‘primitivisms’, modernisation projects and socialist revolutions (Jakovljevic 1967). Jakovljevic and his colleagues functioned in an explicitly anti-colonial explanatory framework, which aimed to eliminate hierarchical political relations between Europe and the decolonising world. As mentioned in the introduction, the political and cultural ‘in-betweenness’ of East European psychiatrists demonstrated very well the instability of the categories of ‘West’ and ‘non-West.’ (Lebow et al. 2019). It was their ambiguous geopolitical position that made this original intervention possible—they did represent the ‘white’ European civilisation, but their own peripheral position within Europe enabled them to promote a unique platform of political and psychiatric solidarity with the decolonising world. Just like political activists, anthropologists and artists posited that Africa and the Balkans shared a history of slavery and colonial subordination, socialist cross-cultural psychiatrists insisted on the fundamental comparability of revolutionary situations and ‘revolutionary personalities’ in the two regions (Baker 2018). Jakovljevic in particular emphasised a universal theory of the relationship between socio-cultural environment and individual psychopathology, which was a result of his cross-cultural research and observations in Guinea and Yugoslavia in the 1950s and 1960s (Jakovljevic 1984).

For socialist psychiatrists, reductive cultural explanations of differences in psychological experiences and perceptions were uncommon—instead, because of their ideological background, they maintained a steady focus on social and economic factors and institutions, and this shaped their interpretations to an important degree. In that sense, Jakovljevic concluded that it was not the assumed inherent, unchangeable and culturally determined simplicity of the mind that decided the status of mental health or illness in the African world; it was a complex web of social, political and cultural circumstances. (Jakovljevic 1984: 168). In fact, ‘the complexity or otherwise of abnormal mental structures depended primarily on the nature and composition of the corresponding social institutions, and not on the level of civilisation, as it is commonly assumed,’ and Jakovljevic argued that even ‘primitive’ cultures could often develop highly intricate sets of social relations (Jakovljevic 1984: 166). This focus on sociological rather than exclusively cultural interpretations of mental distress was, therefore, much more in line with the above-mentioned conclusions of African and Asian transcultural psychiatrists than with those of Jakovljevic’s West European colleagues.

### Questioning Universalism

Another important approach to the intellectual conundrum regarding the role of cultural factors and symbols in psychiatric and psychotherapeutic work was famously espoused by French military psychiatrist Henri Collomb and his experimental Fann mental health clinic in Dakar, Senegal. Collomb’s clinical and research team, set up in the early 1960s, aimed to initiate a truly ground-breaking inter-disciplinary collaboration between transcultural psychiatrists, medical anthropologists, philosophers, ethnologists and anthropological psychoanalysts (Kilroy-Marac 2019). What set this group of practitioners and researchers apart was their openness to local cultural psychotherapeutic systems, and to exploring local systems of practices and beliefs about psychological pathology and healing. Their theoretical framework and methodology were by necessity complex; they were also constantly plagued by intellectual tensions between universalist epistemological systems such as psychoanalysis and cultural relativist approaches. Collomb and his close collaborators opened the clinic to traditional healers and built solid and long-lasting professional relationships with many of them; like Lambo’s Aro village in Nigeria, the Fann hospital combined psychiatric and psychoanalytic clinical practices with some important local social traditions and healing rituals, incorporating and adapting them to the Westernised hospital environment (Kilroy-Marac 2013).

This was perhaps one of the most well-known and far-reaching attempts to establish the discipline of transcultural psychiatry in its most idealistic sense: as a literal communication channel between (West European) clinical psychiatrists and West African traditional healers (Collignon 2018). Moreover, in the early decades of Fann, local cultural interpretations and expressions of mental suffering were not seen as reducible to superficial outer layers of some imagined universal pathology; nor were they immediately (and simplistically) translated into ‘universal’ clinical languages of Western medicine (Kilroy-Marac 2019). At the same time, however, Collomb’s and Fann’s experiment remained riddled with internal tensions over the (in)commensurability of these different approaches and traditions. The Fann school was unique in the postcolonial psychiatric context in that it probed the theory of universality most explicitly and from a decidedly anti-colonial perspective. Still, the work of Fann’s early clinicians and researchers demonstrated and possibly never resolved the theoretical contradictions inherent in this balancing act, as they struggled with difficulties in defining some minimal common ground which would allow for communication and mutual understanding between different cultural systems.

For instance, in Marie-Cecile and Edmond Ortigues’ seminal work *The African Oedipus*, perhaps the most important contribution of the Fann school, the universal reach and explanatory power of psychoanalytic concepts were interrogated and at times critiqued, and the authors concluded that the Oedipus complex could only be adapted to non-European settings if it was understood in a fundamentally pared down, structural sense (Ortigues 1984). Psychoanalysis was best used as a general guide rather than a fixed interpretive framework (Bullard 2005). Still, it was the Oedipus complex—a core psychoanalytic notion—that crucially determined their understanding of Senegalese social structures and familial relationships. Psychoanalysis still ultimately functioned as the universal psychiatric language of a decidedly European origin which could reportedly be used to interpret other cultures’ symbols, meanings and relationships.

Even though Collomb’s insistence on culture was respectful and egalitarian—Rene Collignon notes that traditional beliefs, practices and psychological problems were interpreted at Fann as ‘neither more nor less complex or ‘exotic’ than those found in western patients’—it was difficult for Fann to overcome the complex relationships between cultural relativism and colonial legacies of racist reifications of cultural difference (Collignon 2018). As Stefania Pandolfo points out in relation to post-colonial Moroccan psychotherapy, ‘the study of culture in general, and of “traditional therapies” in particular, is viewed as carrying the legacy of a psychiatric rhetoric systematically seeking in the culture, and especially in the Islamic religion, the roots of North African psychopathology’ (Pandolfo 2000). This further complicates our understanding of the historical relationship between colonial interpretive frameworks and the ideology of universalism (Mills 2017). Despite the many ways in which universalism—in psychiatry as well as in other fields such as human rights—perpetuated colonial tropes, at this historical moment it could also serve an explicitly anticolonial political function, to provide medical evidence to reaffirm the equality and universal rights of all people whose differences were to be accorded less significance. Even within Senegal’s emerging psychiatric profession, questions emerged in the course of the 1970s over whether such an emphasis on cultural specificities and differences could succeed in liberating West African psychiatry from its colonial legacies: within the long history of colonial psychiatry, one was hard pressed to find clinical conceptualisations in which cultural differences were not explicitly linked to notions of hierarchy and worked into theories of inferiority of colonised societies (Vaughan 1991). Without believing in a universal, thoroughly cross-culturally comparable human psyche, could there be a truly postcolonial psychiatry, and did Collomb’s brand of transcultural psychiatry fundamentally depart from colonial norms?

Moreover, as Kilroy-Marac observed, Collomb’s investment in in-depth cultural explorations seemed to go hand in hand with a particular lack of interest in other aspects of environmental influences on mental health, such as political factors or massive sociological transformations which changed the face of Senegal in the 1950s and 1960s: ‘None of Collomb’s publications … hinted at a political consciousness on his part, nor did they reflect the social transformations taking place as struggles for decolonization erupted across Africa.’ In 1967, Collomb emphasised the importance of both ethnologists and ‘psychosociologists’ in a transcultural research team; however, he mentioned no sociological, economic or political factors, focusing instead on the ‘extremely prevalent’ ideas and systems of persecution, which involved ‘exorcizing by magic action’, ‘attacks by spirits’, ‘traditional religious structures’, ‘attacks by cannibal sorcerers’, etc. (Collomb 1967: 17–19). Moreover, his depoliticized understanding of ‘culture’ as a concept in psychiatric theory and practice made it even easier for some of his opponents to critique his innovations, and to argue that the Fann school of transcultural psychiatry did not really reflect on its links to colonial psychiatry nor on Collomb’s own position within the old colonial system. In her recent book, Kilroy-Marac invites readers to imagine how the clinic might have developed under the leadership of Frantz Fanon, who allegedly sent a letter to Senegal’s President Leopold Sedar Senghor in 1953 expressing interest in the position of its director. Fanon was a pronounced critic of Senghor’s brand of Negritude, and it is worth reflecting on how his involvement in the development of Senegalese psychiatry, and his interest in the psychological consequences of political oppression would have transformed the history of the discipline (and of the definition of Senegalese modernity as a whole) (Kilroy-Marac 2019: 106–107). It is indeed intriguing to imagine how the work of the Fann clinic would have advanced if its terms of engagement with the outer world and Senegal’s own history were less depoliticised, less concerned with cultural traditions and more infused by Fanon’s radical and critical political consciousness. Kilroy-Marac’s brief reflection on Fanon’s unanswered letter to Senghor puts Collomb’s legacy of cultural relativism in sharp relief.^11^

### Dangers of Cross-Cultural Encounters

Cross-cultural encounters remained one of post-colonial transcultural psychiatry’s core pre-occupations: the discipline was conceived as both a facilitator of inter-cultural exchanges and a field which dealt with potentially disruptive consequences of cross-cultural encounters and clashes. Early twentieth-century colonial psychiatrists argued that exposing African natives to Westernising influences such as education or urbanisation would have negative psychological consequences, and ‘primitive’ non-Europeans were racially, culturally and biologically unsuited to such alien cultural endeavours (Carothers 1948). Post-colonial transcultural psychiatry, on the other hand, defined the encouragement of cross-cultural dialogue as one of its core professional and political aims. In the new highly interconnected postwar world, global psychiatry sought to radically reconsider the nature, necessity and possible consequences of intense transcultural connections, and to offer a framework for understanding cross-cultural communication and experiences. The very concept of universal humanity served to smooth the progress of inter-cultural dialogue and tolerance.

However, the colonial conceptualisations of cultural clash left long-lasting legacies, and postwar psychiatry continued to treat cross-cultural exposures as well as cultural hybridity as likely causes of psychological breakdown and mental conflict. In fact, different aspects of Carothers’ interpretation of the dangers of ‘detribalisation’ reverberated long after the end of colonial rule. For instance, in the 1950s and 1960s, a large number of leading UK psychiatrists and psychologists accepted that West African immigrants experienced particularly high rates of mental disturbances compared with the native population and immigrants from more ‘advanced’ territories. In the view of these psychiatrists, the core reason for such unusually frequent mental breakdowns was the Africans’ inability to adjust to the ‘modern lifestyles of European civilisation’—the mere exposure of such minds to the complexities of civilisation and to the’orthodox European standards’ increased those patients’ fragility and threatened their mental balance. The UK response to this was even more in line with late colonial explorations: in the 1950s and 1960s, both the British authorities and many psychiatrists agreed that deportations of West African immigrants with mental health issues were the optimal solution. As Matthew Heaton aptly termed it, this practice in fact amounted to ‘the reinforcement of geographical and cultural barriers between the races’, and gave expression to many psychiatrists’ anxieties about the possibility of cross-cultural communication (Heaton 2013).

Lambo and his Nigerian colleagues passionately rejected these arguments, insisting that improved cross-cultural understanding and support, rather than repatriation of mentally ill Nigerians, should be the way forward. Despite such critical voices, the 1959 WMHF’s conference on social change and mental health in Africa saw both West European and African psychiatrists agreeing that the rapid and accelerating Westernisation of the cultural structures in African societies brought with it extreme psychological challenges, to which the WMHF needed to respond immediately (WFMH 1959). Ari Kiev also stated that ‘while there has been little doubt about the relationship between social factors and mental illness, only in the case of Westernization of non-Western peoples has there been unanimous agreement about the effects of social phenomena on the increase of mental illness’ (Kiev 1972). In other words, ‘acculturation’ appeared to be dangerous only if it occurred across the North–South divide, and many in the West appeared to react to it by reinforcing divisions and separation.

Many global psychiatrists of the mid-twentieth century thus saw cultural incompatibilities and clashes in the individual psyches of the ‘detribalised’ or the ‘acculturated’ as the core obstacles to successful transcultural exchange and to preventing mental illness in the developing world. As early post-colonial psychiatry often ignored sociological, economic and political factors, the assumed magnitude of cultural differences took centre stage. One notable exception came out of a productive collaboration between Lambo and Cornell University’s famed psychiatrist Alexander Leighton. Leighton’s background was firmly in social psychiatry, and he and his research group were explicitly concerned with the effects of ‘rapid social changes … on individual and group functioning in both our society and in the under-developed parts of the world.’^12^ This approach was exceptionally important precisely because it was comparative: it assumed, in contradistinction to Kiev, Carothers or Leff, that significant changes in socio-cultural mores affected Western societies in similar ways as they did the decolonising world, and placed Western and non-Western communities within the same framework of ‘acculturation’ (Leighton 1959). Leighton and Lambo’s Cornell-Aro Mental Health Research Project, which began in 1961, aimed to compare conceptualisations and rates of psychological distress among the Yoruba people in Nigeria and rural Canadians in the Stirling County. The researchers concluded that ‘prevalence of psychiatric disorder is associated with [social] disintegration rather than with cultural change as such.’ Mental illness was ‘brought about by disruption in human social networks rather than as the clash of cultures within an individual psyche’ (Heaton 2013: 68). The US-West African research team thus de-exoticised the Nigerian community and the very concept of cultural difference, and made Western and non-Western expressions of mental distress truly comparable—but primarily in the context of social psychiatry.

It was from the East again that some of the most important challenges to this thesis stemmed. Marxist psychiatry developed theories of abrupt social, political and cultural change which went beyond the pathologisation of cultural clashes, and viewed revolutionary transformations in the decolonising world from a significantly more nuanced perspective. For East European experts, while modernisation carried with it a possible increase in psychological suffering and disorders, it was not a failed mission: even ‘primitive’ African inhabitants could adapt to a more technically and culturally advanced surrounding. The socialist rendering of transcultural psychiatry predicted ultimately positive outcomes of the momentous social and political transformations of sub-Saharan Africa (as well as, by extension, the socialist bloc). Cultures and societies could, under propitious circumstances, make revolutionary leaps in their own development and progress: according to Vladimir Jakovljevic, ‘our experiences have clearly demonstrated that a primitive personality, who is young and capable enough, can successfully integrate in a technically and culturally developed environment, even though that integration might be accompanied by temporary mental disorders’ (Jakovljevic 1984: 167).

Moreover, Jakovljevic criticised those interpretations of mental illness which disregarded the creative and revolutionary potential of conflicts between individuals and their social environment: ‘socially caused conflicts might constitute a progressive factor in the development of a society’ and lead to ‘revolutionary resistance against the social organisation or structure’ (Jakovljevic 1984). Mental pathology developed if (reactionary) individuals clashed with the cultural and social norms of a progressive society, but if a similar conflict occurred in a reactionary setting, it was not necessarily a sign of an abnormal personality. If the person in question could not adapt to the anachronistic or ‘decadent’ demands of a non-progressive cultural environment, such conflicts could be constructive or progressive in a revolutionary sense. In fact, such intra-psychic conflicts need not lead to mental illness at all, and might even result in the ‘growth of personality and society, which happens precisely as a consequence of ever more complex internal conflicts and new solutions built into that society by the personality [in question].’ On the other hand, ‘absolute social adaptation to anachronistic and obsolete forms of sociability would necessarily impoverish and alienate the individual’, and possibly push them into difficult neurotic disorders (Jakovljevic 1959: 76–77). The creative potential embedded in social and political conflicts in certain types of societies, therefore, allowed both Eastern Europe and Africa to turn possible psychological disorders into productive and progressive political behaviour. Rapid cultural change, or cultural ‘clashes’ within Westernising African societies, could thus produce unique ‘non-conformist’ personalities, whose social and political contributions would be irreplaceable, and who would be psychologically the healthiest and most stable citizens in the developing world.

Moreover, it was only in Eastern Europe that the notion of ‘acculturation’ in the specific context of decolonisation and rapid globalisation was interpreted in an explicitly positive way. For Yugoslav commentators, psychiatrists and anthropologists, for instance, it was of foremost importance to insist on the dynamic nature of African cultures and societies: writing against the idea that (sub-Saharan) African societies were static, ahistorical and marked by ‘unchangeable traditions and tribal exoticism’, Jakovljevic’s anthropologist colleague Biserka Cvjeticanin emphasised their dynamic history and their current creative grappling with large-scale changes. Before colonial conquests, during colonial regimes and after decolonisation, different African groups and societies experienced constant change both within the confines of their own borders and in contact with other cultures, so that their capability of dynamic developmentwas in no fundamental way different from that of Western societies (Cvjeticanin 1979).

Cvjeticanin introduced Marx’s definition of acculturation—as a process of original ‘creation under the pressure of novel circumstances and by no means a simple dissolution of a culture which suffered a blow from outside’ (Cvjeticanin 1979: 789). Moreover, Cvjeticanin was one of the very few voices who insisted that acculturation was a two-way process, which changed Western societies as well. It was no coincidence that such a voice came from Marxist Eastern Europe: this intervention meant that the influence of African (and, by extension, other non-Western) cultures on the Western world was not minuscule. Moreover, Cvjeticanin warned that acculturation was not assimilation or a mechanical transplantation of certain traits and mores from a more developed society to a less developed one, but it meant ‘transformation and creative integration.’ In the course of this process, the receiving culture demonstrated its own dynamism and ability to not only adopt new elements but also change them and ensure the authenticity and continuity of its own identity. In that sense, African cultures were neither mere imitators of more developed traditions nor unchangeable/ahistorical: just like all other societies, they chose which foreign elements and cultural aspects to adopt, adapt and fit in their own existing structures, thereby producing novel (dynamic and modern) creative totalities. In this reading, the process of acculturation was fully divested of any potentially pathological effects or meanings, and non-Western social systems were actively de-exoticised.

Therefore, in line with Jakovljevic’s arguments, Cvjeticanin did not see cross-cultural exchange as necessarily disastrously disruptive—while not without problems or conflicts, rapid socio-cultural change could lead to productive situations and progress. African societies were, therefore, not exhausting all their energies merely trying to catch up, while experiencing extraordinary levels of mental distress as a consequence. They were producing authentic cultural contributions in the process of their fast development and transformation.

## Conclusion

As Aubrey Lewis noted, the emerging discipline of global psychiatry had high hopes for its own professional and political status: ‘Many psychiatrists believe they have a knowledge of the forces of human nature, in individuals and in groups, which entitles them to take a large part in … advising on human relations between people and even between communities and nations.’^13^ As this article has demonstrated, transcultural psychiatry was an essential part of the post-1945 search for stable peace, and consciously fashioned itself as a facilitator of cross-cultural communication and understanding in the context of postwar reconstruction and decolonisation. It grappled with notions of ‘cultural difference’ in order to re-define the relationship between race, culture and individual psyche, and to move away from the difficult legacies of colonial psychiatry. Importantly, transcultural psychiatry rose to prominence hand in hand with social psychiatry, and both sub-disciplines sought to explore the role of environmental, socio-cultural factors in the aetiology and development of mental illness. But even though they were theoretically very close, the two disciplines developed in significantly different directions, as transcultural psychiatry focused on researching patient populations from the former colonies, grappled with the lingering influence of colonial interpretive frameworks and ultimately defined aetiologically relevant environmental factors in narrowand exoticising terms. In the first decades after the Second World War, this had an effect of further reifying the concept of ‘cultural differences’ between European and non-European populations, and, paradoxically, undermined the notion of psychiatric universalism, championed by some of the most influential cross-cultural psychiatrists.

The article has explored a variety of internal contradictions which complicated the theory of universal human psychology. Leading postwar psychiatrists denied the importance of cultural differences for understanding cross-cultural mental pathology but nevertheless focused primarily on narrowly conceived ‘cultural factors’—the ‘study of racial, cultural and geographical determinants’ and ‘ethnic particularities’– to the exclusion of broader social, economic or political categories, which could have facilitated comparisons between Western and non-Western patient populations (Margetts 1960: 453). Before the onset of a more anthropologically oriented cross-cultural psychiatry in Western Europe, it was clinicians from the decolonising world and from socialist Eastern Europe who responded to these problems in innovative ways, grappling with the afterlives of colonial interpretive frameworks within the theory of universality, and developing nuanced alternatives in order to overcome the binary of Eurocentric universalism and extreme cultural relativism.

Many of these debates remain relevant in contemporary clinical practice. In their critique of the idea of cultural competency, Arthur Kleinman and Peter Benson identified some very similar trends in present-day psychiatric clinics, where ‘cultural competency’ is imagined as a technical set of skills that can be acquired relatively easily precisely because ‘culture’ is defined as static, homogeneous and isolated, and regularly made identical with ethnicity or race. Moreover, clinicians often seem to see ‘culture’ as separate from broader political, social and economic concerns, and focus on such narrowly defined cultural differences to the exclusion of patients’ other experiences or traits (Kleinman and Benson 2006). The current reductive interpretation of ‘culture’ in psychiatric clinical practice likely has roots in the post-war history of psychiatric universalism. Understanding this history can thus shed light on ways to critique such reductionism, and help develop more nuanced frameworks for exploring patients’ complex social worlds and their interaction with psychological illness.

## Footnotes

1 Aubrey Lewis, ‘Research in social psychiatry’, 1959, Archive of King’s College Institute of Psychiatry, Personal papers of Aubrey Lewis. IOP/PP3/5/13, 1-6; see also Lewis, ‘Modern trends in psychiatry’, 1954, IOP/PP3/5/15.

2 Following pioneering scholars such as Alice Bullard, Matthew Heaton and Jatinder Bains, I use the term ‘transcultural psychiatry’ to refer to the international movement as well as a set of institutions in global psychiatry, which emerged in the 1940 s and 1950 s as part of the transition from colonial to post-colonial psychiatry. This new discipline primarily aimed to ‘bridge [cultural] differences in beliefs and practices related to spirit, psyche, healing and wellness’, and to create a coherent framework for cross-cultural comparisons of psychological illnesses (Bullard 2007). It also sought to provide a platform for communication between different cultural interpretations of mental pathology (Heaton, 2013:64-65).

3 Western colonial regimes were certainly not homogeneous or identical, and there were important local specificities and historical differences between colonial powers in forms of rule, imperial rhetoric, aims and motivations (Toye and Thomas 2017). Different colonial situations, of course, produced historically specific and locally shaped bodies of medical and psychiatric knowledge, which often changed quite radically over time (Linstrum 2016). But leading scholars in the field have long used the concept of ‘colonial psychiatry’ to refer to the collective set of theories, institutions and clinical practices produced by West Europeans about non-European colonial subjects during the colonial period. This is not to argue that there were no notable differences between different schools of colonial psychiatry, but simply to acknowledge that this composite profession shared important characteristics across the board, such as racialized hierarchical frameworks, evolutionary thinking about non-Europeans as inhabiting a ‘childhood stage’ of civilisation, or belief in fundamental differences between the European and ‘primitive’ minds (Vaughan 1991; McCulloch 1995; Keller 2007).

4 Even so, he received official support from the WHO, as well as from Margaret Mead. ‘Carothers’ study on mental health in Africans’, Archive of the WHO, M4/445/13, 1953.

5 The notion of ‘acculturation’ referred to the process of exposing African natives to Westernising influences such as education or urbanisation, and was closely linked to the equally loaded concept of ‘detribalisation’. Carothers argued that such an introduction of Western culture upset the Africans’ ‘equilibrium’ and had negative psychological consequences because 'primitive' non-Europeans were racially, culturally and biologically unsuited to more ‘advanced’ cultural values and mores (Carothers 1948: 197–219).

6 The Ciba Foundation is a research and educational charity organisation, established by the Swiss pharmaceutical company CIBA Limited (now Novartis). Since 1947, the Ciba Foundation has been supporting transnational interdisciplinary medical and biological research, mainly through organising cross-disciplinary workshops and symposia. The above-mentioned 1965 symposium was the Foundation’s first one in psychiatry, and proved to be one of the most important and ground-breaking events in the field of transcultural psychiatry, not least because its participants included almost all leading practitioners and researchers from around the world.

7 This theory is well summarised by Carothers’ statement that ‘the African way of life is, with minor variations, the way that all mankind, including our own ancestors, is likely to have followed in the past’ (Carothers 1960).

8 Postwar global and transcultural psychiatry, and the related WHO projects such as the IPSS, were heavily dominated by British and North American psychiatry, while the French school was increasingly sidelined, and it developed in a less universalist direction of ‘ethnopsychiatry’, fundamentally influenced by ethnologist George Devereux (Bullard 2007). The reasons for excluding French psychiatrists from large comparative projects such as the IPSS are not entirely clear. French experts were initially involved in planning a range of WHO-led projects in global psychiatry and psychiatric epidemiology, but eventually exerted much less global influence than their UK, US and even Scandinavian colleagues. In his recent book, Harry Wu offers a (fairly limited) glimpse into the problem, by quoting US psychiatrist Paul Lemkau, a very influential figure in WHO mental health work, who reportedly thought that French scholars were simply ‘not good enough’ for this type of research (Wu 2021:51).

9 Leff received criticism from the psychiatric side as well; Raymond H. Prince, Canada’s leading transcultural psychiatrist and Professor of Psychiatry at McGill University, anticipated some of the anthropological concerns about Leff’s theory of languages of emotion in his critical review, noting that ‘Leff appears to be reverting to a kind of primitive-civilized dichotomy reminiscent of nineteenth-century views of cultural evolution’ (Prince 1983: 117–118).

10 Archive of the University of Ibadan (Faculty of Medicine), COMED 10-20-31, Request for Grant from WHO, Geneva, January 1965.

11 Frantz Fanon’s was, of course, the most influential critique of the colonial psychiatric project and its harmful political and psychological consequences. His incisive analyses of the psychopathology of colonialism went well beyond the transcultural psychiatric discussions about universalism, cultural difference and ethnocentrism, focusing on much broader issues, whose significance could not be done justice in the article. In many ways, Fanon was outside the regular knowledge networks of transcultural psychiatry, an outsider who was larger than the profession itself, although his clinical work certainly led him to engage in profound ways with the relevance of cultural traditions in psychotherapy, and to critique the harmful effects of imperially driven psychiatric universalism (see Robcis 2020).

12 The Cornell Programme in Social Psychiatry, 1st Annual Report, 30 June 1959, 15.

13 Lewis, ‘Modern trends in psychiatry’.
